# Fatigue in Post-COVID-Condition is accompanied by hypoperfusion of right-occipital areas

**DOI:** 10.1371/journal.pone.0335038

**Published:** 2025-11-21

**Authors:** Jonas A. Hosp, Nils Schröter, Marco Reisert, Sibylle Frase, Elias Kellner, Hansjörg Mast, Cornelius Weiller, Horst Urbach, Alexander Rau

**Affiliations:** 1 Department of Neurology and Clinical Neuroscience, Medical Center – University of Freiburg, Faculty of Medicine, University of Freiburg, Freiburg, Germany; 2 Department of Diagnostic and Interventional Radiology, Medical Center – University of Freiburg, Faculty of Medicine, University of Freiburg, Freiburg, Germany; 3 Department of Stereotactic and Functional Neurosurgery, Medical Center - University of Freiburg, Faculty of Medicine, University of Freiburg, Freiburg, Germany; 4 Department of Neuroradiology, Medical Center – University of Freiburg, Faculty of Medicine, University of Freiburg, Freiburg, Germany; 5 Freiburg Brain Imaging Center, Medical Center – University of Freiburg, Faculty of Medicine, University of Freiburg, Freiburg, Germany; Museo Storico della Fisica e Centro Studi e Ricerche Enrico Fermi, ITALY

## Abstract

**Background and purpose:**

A proportion of individuals recovering from COVID-19 continue to experience persistent symptoms, including fatigue and cognitive difficulties — a syndrome commonly referred to as Post-COVID condition (PCC), which affects an estimated 2–10% of cases. In this study, we evaluated cerebral blood flow (CBF) to better understand the pathophysiological mechanisms underlying PCC.

**Materials and methods:**

In this prospective, monocentric study, we analyzed clinical and cerebral blood flow (CBF) data from a cohort of 55 patients who met the WHO diagnostic criteria for Post-COVID condition (PCC) and underwent MRI approximately 11 months after a positive PCR test for SARS-CoV-2. These PCC patients were compared to a matched control group of 36 individuals who had contracted COVID-19 but did not develop PCC. CBF was assessed using arterial spin labeling (ASL), a promising non-invasive technique that provides high spatial resolution for quantifying cerebral blood flow. Additionally, we examined changes in gray matter volume and atrophy using FreeSurfer-based cortical morphometry. We further explored the relationship between regional CBF alterations and clinical symptoms, including cognitive and olfactory function, as well as fatigue.

**Results:**

In our cohort, 59% of PCC patients could not return to their previous level of independence or employment due to symptoms, and 81% reported fatigue on the WEIMuS questionnaire. Conventional MRI showed no evidence of cortical atrophy. While no significant differences in regional CBF emerged after FDR correction, a more explorative threshold (p < 0.005) revealed reduced CBF in the right angular and middle occipital gyri in PCC patients. Fatigue, as assessed by the WEIMuS, was significantly correlated with reduced CBF in the right occipital regions, particularly for physical fatigue, but no associations were found with cognitive or olfactory performance.

**Conclusion:**

In PCC patients, fatigue was associated with reduced perfusion in right-sided occipital regions, suggesting a potential pathophysiological basis for this symptom. These findings may also provide an imaging biomarker to aid in the diagnosis of PCC.

## Introduction

According to World Health Organization criteria, Post-COVID Condition (PCC) is diagnosed when individuals with a history of confirmed or probable SARS-CoV-2 infection experience persistent symptoms—such as fatigue or cognitive difficulties—that impair daily functioning for at least two months and begin no sooner than three months after the acute illness [1]. Based on this definition, a total of 2–10% of COVID-19 survivors develop PCC [2,3]. This frequently progresses into a chronic condition, as 85% of patients reporting complaints two months after COVID-19, still reported symptoms – primarily fatigue, persistent impairment of olfaction and deficits in attention and memory occur – one year after initial symptom onset [4,5]. A substantial portion of individuals with PCC report ongoing limitations in their professional and personal lives, with estimates suggesting that up to 39% experience work-related restrictions, highlighting the condition’s broader societal impact [6]. Nonetheless, the pathophysiology underlying neurocognitive deficits is poorly understood and established biomarkers are lacking.

Arterial spin labeling (ASL) is an MRI-based method for assessment of cerebral blood flow (CBF) by magnetically labeling protons in inflowing arterial blood prior to their entry into brain tissue [7]. Thus, ASL provides quantifiable CBF-maps in a few minutes, without relying on exogenous contrast agents [8]. As there is a strong association between local CBF and glucose metabolism in the brain [9], ASL is an established measure of functional integrity and the physiological state in patients with epilepsy [10], neurodegenerative [11,12] or neoplastic diseases of the brain [13]. Although the ASL technique has already been used to study brain changes in COVID-19 survivors [14,15], data from patients diagnosed with Post-COVID condition according to the WHO definition are sparse. ASL was utilized to reveal an association between impaired olfaction and orbitofrontal hypoperfusion [16] and in a “long COVID” cohort that was not defined according to WHO criteria, a link between reduced CBF in left frontal-temporal gyrus with both fatigue and worse cognition [17].

Motivated by these findings, we investigated CBF using ASL in a prospective monocentric cohort of PCC patients diagnosed according to the WHO criteria. PCC patients were compared to a cohort of subjects that had contracted COVID-19 without lasting symptoms. We further investigated the association between regional CBF profiles and clinical symptoms, i.e., cognitive and olfactory performance or fatigue.

## Methods

### 1. Study participants and clinical outcomes

We report data from a monocentric, prospective cohort of 55 patients (median age 39 [23] years; range: 19–70 years; 37 females), who were admitted to the outpatient clinic of the Department of Neurology and Clinical Neuroscience of the University Hospital Freiburg between June 16^th^, 2020 and October 6^th^, 2022 due to neurocognitive symptoms in the chronic phase after a COVID-19 infection. This cohort has a substantial overlap with a previously published study that revealed widespread microstructural alterations in both COVID-19 survivors and patients with PCC [18]. The local ethics committee approved this study (EK 211/20) and all subjects provided written informed consent in accordance with the Declaration of Helsinki and its later amendments. Inclusion criteria were: 1) reverse transcription polymerase chain reaction (rt-PCR) confirmed SARS-CoV-2 infection; 2) meeting the diagnostic framework outlined by the World Health Organization (WHO) for Post-COVID Condition, which includes symptoms persisting for more than two months and starting at least three months after the initial infection [1]; 3) execution of a cranial MRI without artifacts hampering the evaluation of ASL. Exclusion criteria were any pre-existing neurodegenerative disorders and age < 18 years. A collective of 36 individuals (median age 44 [22.5] years; range: 25–62 years; 24 females) in the chronic phase after PCR-confirmed COVID-19 infection who did not develop PCC or report persistent symptoms served as control group. PCC-patients and subjects of the control group were examined and surveyed by board-certified (JH and SF) or experienced (> 7 years of training, NS) neurologists, including a detailed patient history and assessment of current neurological symptoms as self-reported.. The degree of current disability was graded as follows: 0, no relevant restrictions; 1, relevant restrictions but able to work; 2, reduction of work quota necessary; 3, inability to work and/or restriction of daily life activities. Disease severity during the acute stage was scored according to a modified version of the German definitions [19]: 1, no signs of pneumonia; 2, pneumonia, outpatient treatment; 3, pneumonia, inpatient treatment; 4, acute respiratory distress syndrome (ARDS), endotracheal ventilation at intensive care unit (ICU). Disease severity was considered to be “mild” in case of outpatient treatment (i.e., 1–2) and as “severe” in patients that required hospitalization (i.e., 3–4). Cognitive functions were assessed with the German version of the Montreal Cognitive Assessment (MoCA version 7.1, www.mocatest.org) [20]. The highest possible global MoCA score is 30 with higher scores indicating better performance, the cut-off score for cognitive impairment was defined as < 26 [20]. Correction for years of education (YoE) was performed (+1 point in case of ≤ 12 YoE). Fatigue was evaluated using the Würzburg Fatigue Inventory in Multiple Sclerosis (WEIMuS) [21], a self-rating questionnaire for symptoms of physical and cognitive fatigue. In addition, the Geriatric Depression Scale-15 (GDS) [22] was surveyed. Olfaction was assessed using Burghart-Sniffin’-Sticks® (Burghart Messtechnik GmbH, Wedel, Germany) (normosmia: 11–12 correctly identified odors; hyposmia: 7–10 correct odors; anosmia: ≤ 6 correct odors) [23]. Ammonium was used to assess trigeminal function.

### 2. Cerebral MRI

#### MRI acquisition and calculation of cerebral perfusion parameters.

MRI was performed with a 3 Tesla scanner (MAGNETOM Prisma, Siemens Healthcare, Erlangen, Germany) with a 64-channel head and neck coil. T1‐weighted (T1w) images were acquired with a three‐dimensional (3D) magnetization‐prepared 180° radio‐frequency pulses and rapid gradient‐echo (MP‐RAGE) sequence (repetition time: 2500 ms, echo time: 2.82 ms, flip angle: 7°, TI = 1100 ms, GRAPPA factor = 2, 1.0 mm^3^ isotropic voxels, 192 contiguous sagittal slices). Time-encoded pseudo-continuous ASL (pCASL) was acquired with a sequence based on the free lunch approach with Hadamard-encoding [24] and background suppression. Thirty-two 3-mm slices were acquired with FOV = 256 mm, matrix size = 128 × 128, TE/TR = 22.5/4500 ms, GRAPPA = 2. The labeling plane was positioned inferior to the center of the imaging slab with a labeling duration of 1800 ms and post-labeling delay (PLD) of 200 ms. For each participant, the labeling plane offset of the pCASL sequences was adjusted (90–125 mm from the center of the imaging slab) to ensure the vessels were perpendicular to the labeling plane [25]. Two equilibrium magnetization images (M0) with right-left and left-right phase-encoding were acquired to convert perfusion-weighted images into physiological units of blood flow. Data processing was implemented on a local instance of the post-processing platform NORA (www.nora-imaging.org). Acquired data was motion corrected using SPM12 (Wellcome Centre for Human Neuroimaging, London, UK). Data was then decoded according to the Walsh-Matrices [26]. With the decoded data and the M0 maps, absolute CBF maps were calculated using the Oxford ASL toolbox (https://asl-docs.readthedocs.io/en/latest/oxford_asl.html) including Bayesian Inference for Arterial Spin Labelling MRI (BASIL) [27]. Subsequently, CBF maps were normalized by dividing each voxel’s value with the mean CBF of the patient’s whole brain. T1w imaging datasets were automatically segmented into white matter, gray matter and cerebrospinal fluid (CSF) using CAT12 (http://www.neuro.uni-jena.de/cat/) and CBF images were co-registered to the T1w images. Validity of co-registrations between ASL images and T1w-derived tissue probability values (TPV) were manually confirmed. Further quality control was performed by visually inspecting each individual CBF map and CAT12 segmentation. The diffeomorphic warp was used to transfer the quantitative normalized CBF maps to the Montreal Neurological Institute (MNI) space. Here, CBF values were read after parcellation according to the Automated anatomical labelling atlas 3 (AAL3) [28]. The FreeSurfer pipeline was employed to obtain information on cortical thickness, surface area, and gray matter volume. Derived data was parcellated according to the Desikan-Killiany atlas (DKT) [29].

### 3. Statistical analysis

Statistical analyses were performed using R (https://www.R-project.org/) and SPSS version 25 (IBM, Ehningen, Germany). Shapiro-Wilk test was used to assess normal distribution of data. In case of normal distribution, data were indicated as mean (standard deviation) and t-tests were used for group-comparisons. If data were not normally distributed, data were indicated as median [inter quartile range] and nonparametric Mann-Whitney-U-tests were applied. Correlations between clinical data were assessed with Spearman’s rank correlation test, Bonferroni-correction was applied to account for multiple testing. We assessed group-level CBF differences using analysis of covariance (ANCOVA), adjusting for age and sex as confounding variables. The False Discovery Rate (FDR) was applied to correct for multiple comparisons. To investigate associations between the CBF in PCC-patients and clinical scores, we employed mixed linear models while controlling for “age” and “sex” and subsequent FDR-correction to account for multiple comparisons.

## Results

We report MRI data of a cohort of 55 patients (age 39 [23] years; range: 19–70 years; 37 females) fulfilling the WHO`s diagnostic criteria for Post-COVID-Condition (PCC-group). This sample is a subset of a previous publication on microstructural cerebral alterations in individuals with PCC [18]. Demographics and clinical characteristics are listed in **Table 1** and **Table 2**. Neurological examinations revealed no focal deficit. Patients most frequently reported fatigue, attention and memory disturbances, and difficulties with language and multitasking—symptoms which significantly impacted their cognitive functioning. Regarding MoCA performance, 17 of 54 patients (31%) performed below the cut-off score for cognitive impairment [20]. Olfactory performance was impaired in 31 of 55 patients (56%). 43 of 53 patients (81%) revealed overall symptoms of fatigue in WEIMuS. On a subscore level 77% (n = 41) were above the cutoff for mental and 72% (n = 38) above the cutoff for physical fatigue. The GDS-15 indicated no relevant level of depression in the PCC-cohort on group-level, although 13 patients (25%) exceeded the cut-off value. Between the parameters current disability, disease severity, MoCA-performance, WEIMuS, olfactory performance, and GDS-15, the following significant associations were present: WEIMuS was correlated with current disability (*P* < 0.001; Spearman’s Rho: 0.78) and GDS-15 (*P* < 0.001; Spearman’s Rho: 0.76) and inversely with olfactory performance (*P* = 0.004; Spearman’s Rho: −0.38). In addition, current disability was associated with GDS-15 (*P* < 0.001; Spearman’s Rho: 0.63). Furthermore, 36 individuals (age 44 [22.5] years; range: 25–62 years; 24 females) with passed COVID-19 infection but without lasting subjective impairment were enrolled as control group. Detailed demographic and clinical information is provided in **Tables 1** and **2**. Compared to the PCC-group, no significant differences were present for age, sex, the delay between positive PCR and imaging, severity of acute COVID-19 and MoCA-performance (all *P *> 0.05). Regarding clinical readouts, controls performed significantly better in olfactory testing (Mann-Whitney-U, *P* = 0.01) and were significantly less affected in GDS-15 (Mann-Whitney-U, *P* < 0.001) and WEIMuS (t-test, *P* < 0.001). Cortical morphometric analyses using FreeSurfer [30]did not indicate measurable volume loss or thinning across cortical regions when compared to controls. Moreover, we did not note a significant association of structural parameters with PCC-related symptoms (i.e., MoCA-performance, olfaction, and WEIMuS scores). In summary, structural MRI did not reveal alterations explaining the neurological symptoms.

**Table 1:** Demographics and comorbidities of study participants.

	Post-COVID-Condition (PCC; n = 55)	Control Group (n = 36)	P-value
**Demographic data**	**n (%) or median [IQR]; range**	**(%) or median [IQR]; range**	
**Age (years)**	39 [23]; 19–70	44 [22.5]; 25–62	**0.95** ^**1**^
**Sex (male/ female)**	18 (32)/ 37 (68)	12 (33)/ 24 (67)	**0.69** ^**2**^
**Δ positive PCR - cMRI (days)**	319 [194]; 90–975	227 [175]; 145–943	**0.29** ^**1**^
**Comorbidities**	**n (%)**	**n (%)**	
Adipositas	2 (4%)	3 (8%)	
Asthma/COPD	3 (6%)	1 (3%)	
Coronary heart disease	0 (0%)	1 (3%)	
Diabetes	7 (13%)	0 (0%)	
History of depression	5 (9%)	2 (5%)	
Hypocholesterolemia	3 (6%)	1 (3%)	
Arterial hypertension	6 (11%)	4 (11%)	
Hypothyreosis	6 (11%)	6 (17%)	
Migraine	3 (11%)	1 (3%)	
Obstructive sleep apnoea	1 (2%)	0 (0%)	
Peripheral arterial occlusive disease	1 (2%)	0 (0%)	
Restless legs syndrome	1 (2%)	0 (0%)	
Rheumatoid arthritis	1 (2%)	0 (0%)	

**1 Mann-Whitney-U test; ^2^ X^2^-test.**

**Table 2:** Clinical scores of study participants.

	Post-COVID-Condition (PCC; n = 55)	Control Group (n = 36)	P-value
	median [IQR]; range; n outside of norm (%)	median [IQR]; range; n outside of norm (%)	
**Disease severity score** ^**1**^			
**Mild course of COVID-19**	52 (95%)	35 (97%)	**0.53** ^**2**^
**1**	39 (71%)	23 (64%)	
**2**	13 (24%)	12 (33%)	
**Severe course of COVID-19**	3 (5%)	1 (3%)	
**3**	3 (5%)	1 (3%)	
**4**	0 (0%)	0 (0%)	
**Grading of current disability** ^**2**^			**< 0.001** ^**2**^
**0**	0 (0%)	36 (100%)	
**1**	23 (41%)	0 (0%)	
**2**	10 (19%)	0 (0%)	
**3**	22 (40%)	0 (0%)	
**Current neurological symptoms**			
Impaired attention	55 (100%)	0 (0%)	
Impaired concentration	55 (100%)	0 (0%)	
Memory impairment	55 (100%)	0 (0%)	
Impaired multi-tasking Word-finding difficulties	54 (98%) 46 (84%)	0 (0%) 0 (0%)	
Fatigue	54 (98%)	0 (0%)	
**Clinical readouts** MoCA sum score (corrected for years of education; norm ≥ 26/30)^3^	27 [3]; 20–30; 17 (31%)	27 [3]; 23–30; 7 (19%)	**0.06** ^**4**^
Correct perception of smell (norm ≥ 11/12)	10 [2]; 0–12; 31 (55%)	11 [2]; 8–12; 14 (39%)	**0.01** ^**4**^
Würzburg Fatigue Inventory in Multiple Sclerosis score	46 [19]; 4–65; 43 (81%)	9 [19]; 0–48; 4 (11%)	**< 0.001** ^**4**^
(WEIMuS; norm: ≤ 33/68)^5^- cognitive fatigue (norm: ≤ 17/36)	25 [12]; 0–36; 41 (77%)	4 [11]; 0–24; 5 (14%)	**< 0.001** ^**4**^
- physical fatigue (norm: ≤ 16/32)	22 [8]; 4–32; 38 (72%)	3.5 [11]; 0–9; 2 (6%)	**< 0.001** ^**4**^
Geriatric Depression Scale (GDS-15; norm: ≤ 7/15)^5^	5 [4]; 1–13; 13 (25%)	1 [4]; 0–9; 1 (2%)	**< 0.001** ^**4**^

^1^Disease severity score: (1) no pneumonia; (2) pneumonia, outpatient treatment; (3) pneumonia, inpatient treatment; (4) ARDS, endotracheal ventilation at ICU; ^2^ Grading of current disability: (0) no relevant restrictions; (1) relevant restrictions of daily life activities but able to work; (2) reduction of work quota necessary; (3) inability to work and restriction of daily life activities; ^3^ data available only for n = 54 patients; ^3^ X² test; ^4^ Mann-Whitney-U test; ^5^ data available only for n = 53 patients.

**Fig 1:**
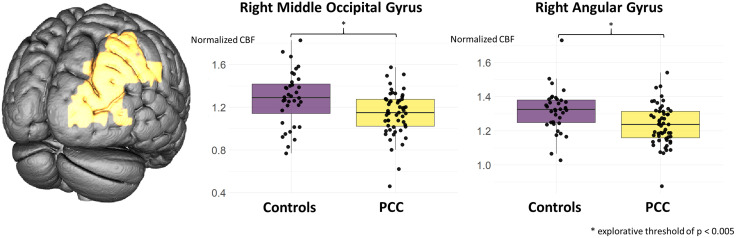
Group comparison of normalized CBF between Post-COVID-condition (PCC) and the control group. An explorative threshold of p < 0.005 revealed group differences (indicated with *) in the right middle occipital and the right angular gyrus (given in yellow).

**Fig 2:**
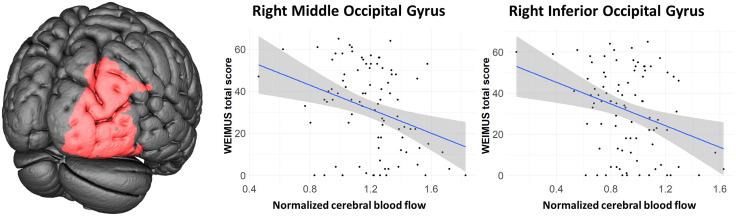
Correlation of normalized CBF with total score in the WEIMuS questionnaire. Areas with significant associations are given in red.

With respect to the whole brain CBF, no group difference was present between PCC-patients and controls (F(1) = 2.13, *P* = 0.13; ANCOVA with nuisance covariates “age” and “sex”). Also in region-wise comparisons using ANCOVAs with nuisance covariates “age” and “sex”, no significant region remained after FDR-correction for multiple comparisons. However, by applying a more liberal and explorative threshold of *P* < 0.005, there seems to be a trend for a CBF-reduction for the right angular gyrus (uncorrected *P *= 0.0015) and in the right middle occipital gyrus (uncorrected *P* = 0.0035; **Fig 1**). To investigate associations between the CBF in PCC-patients and clinical scores, we performed region-wise partial Spearman’s rank correlation tests with nuisance covariates “age” and “sex” and FDR-correction to account for multiple comparisons. Here, significant associations were present between CBF-reduction in the right middle (*P* = 0.043) or inferior occipital gyrus (*P* = 0.043) and the total WEIMuS score (**Fig 2**). With respect to subscores, significant associations were present for physical (right middle occipital gyrus *P* = 0.033; right inferior occipital gyrus *P* = 0.033), but not mental fatigue (right middle occipital gyrus *P* = 0.091; right inferior occipital gyrus *P* = 0.091). No significant associations were present for MoCA- and olfactory performance or the GDS-15 (all *P* > 0.05).

## Discussion

Here, we report clinical and cerebral perfusion data from a well-characterized prospective cohort of patients diagnosed with Post-COVID-Condition (PCC). A significant inverse association was present between fatigue (assessed by the WEIMuS-score) and CBF in the right middle and inferior occipital gyrus. In terms of subscores, this effect was more pronounced when physical rather than mental fatigue was examined. Thus, fatigue is linked to hypoperfusion of right-sided occipital areas in PCC-patients. When compared to a control group of unimpaired individuals who had previously contracted a COVID-19 infection, FreeSurfer-based analysis did not provide any detectable evidence of global or cortical atrophy. Regarding regional CBF, no significant difference emerged between groups after FDR-correction for multiple comparisons. However, when a more explorative threshold was applied (*P* < 0.005), a reduction of CBF was found in the right angular gyrus and the right middle occipital gyrus.

Regional CBF as a measure of tissue perfusion was examined in COVID-19 at different stages and in different populations. Prior imaging in critically ill COVID-19 patients revealed bilateral frontotemporal perfusion deficits during the acute phase. Similar findings were seen in later stages, though evidence remains inconsistent across studies [31]. This pattern was confirmed in a larger cohort of 59 patients that were examined in the subacute phase (in average 3 weeks after infection) after a severe course of disease [32]. However, alterations in functional connectivity revealed by resting state functional MRI (rs-fMRI) in the frontal, temporal, and occipital lobes were still present after 6 months in a group of recovered patients after a moderate to severe course of COVID-19 [33]. Moreover, the feature of hypoperfusion in the (sub)acute phase does not appear to be limited to patients with severe infection, as reduced CBF with a fronto-temporal emphasis was detected in 28 patients with a mild course of disease when compared to controls [34]. Similar to patients with severe disease, a CBF reduction (orbitofrontal cortex and subcortical structures) was still detectable 4−5 months after mild COVID-19 infections [14]. In summary, infection with SARS-CoV-2 appears to lead to long-lasting changes in cerebral perfusion regardless of initial disease severity.

Beyond this, ASL revealed altered cerebral perfusion in PCC compared with healthy controls: In 24 patients with lasting cognitive complaints 6 months after infection, widespread hypoperfusion with emphasis on the frontal cortex was identified [15]. Likewise, ongoing symptoms 4–5 months after COVID-19 were associated with altered functional connectivity between temporal, parietal, occipital and subcortical regions in another study investigating PCC patients [35]. These findings are in contrast to our study, which detected reduced regional CBF only at trend level within the right angular and middle occipital gyrus. However, aforementioned populations were compared with healthy controls who had not previously been infected with SARS-CoV-2. Since the infection alone can cause long-lasting changes in cerebral tissue perfusion with emphasis on frontal lobes (see above), healthy controls are inadequate for differentiating characteristics that are “PCC-specific” since they might be sequelae of the COVID-19 infection itself without a pathophysiological link to PCC. For this reason, we compared perfusion data of PCC-patients with a well-defined cohort of subjects that had contracted COVID-19 without lasting symptoms. Here, PCC-specific differences in regional perfusion were only present using an exploratory threshold. However, a significant correlation between fatigue and hypoperfusion could be detected for the right hemisphere in inferior and middle occipital areas. In line with this finding, reduced occipital perfusion was observed associated with ongoing self-reported fatigue 4 months after mild COVID-19 [14]. Moreover, hypoperfusion of distinct brain regions could be linked to symptoms in PCC, as an association of impaired olfaction was found with orbitofrontal CBF reduction [16], and fatigue could be linked to left frontotemporal brain regions [17] and reduced thalamic oxygen levels [36].

Interestingly, our observation of parieto-occipital hypoperfusion aligns with findings from studies on myalgic encephalomyelitis/chronic fatigue syndrome (ME/CFS) [37]. Here, ASL showed a reduced regional CBF at rest within right-hemisphere occipital regions including the inferior occipital gyrus that was correlated with the level of self-reported fatigue [38]. Moreover, a reduced Blood-Oxygenation-Level Dependent (BOLD)-signal as a measure of tissue perfusion in response to a selective attention test has been described in the right medial occipital cortex [39]. The degree of hypoactivation was also associated with outcomes in physical and mental health questionnaires. In addition to such a reduction of perfusion, voxel-based morphometry revealed reduced grey matter volume in bilateral occipital areas in ME/CFS patients [40] and cerebral proton magnetic resonance spectroscopy detected an increase in choline within the occipital cortex in line with an abnormality of phospholipid metabolism [41]. Moreover, an involvement of the occipital lobe has been described in fatigue in the context of diseases other than ME/CFS: In patients suffering from Crohn’s disease, grey matter volumes in occipital areas were inversely correlated with fatigue scores [42] and significantly lower occipital resting-state activity could be detected in patients with post-stroke fatigue [43]. Finally, the occipital cortex has been shown to be involved in the evolution of task-induced fatigue [44,45]. Physiologically, the right middle occipital cortex is a part of the dorsal visual stream [46] and has a role in spatial visual processing [47] whereas the inferior occipital cortex is part of the ventral visual stream involved in spatial processing and face recognition [48]. Interestingly, fatigue and increased fatigability are well-known phenomena in patients with cerebral visual impairment due to damage of the dorsal and ventral visual stream [49,50]. Here, a compensatory activation and involvement of cortical areas outside of the occipital lobes has been discussed as a possible explanation [50].

A notable limitation of this study is its exclusive focus on cortical morphometry, without investigation of subcortical structures such as the thalamus and basal ganglia. This is particularly relevant given prior evidence of basal ganglia involvement in post-COVID fatigue [51]. While we employed whole-brain cortical morphometry, including assessment of cortical thickness and surface area using FreeSurfer, this method is not feasible in deep gray matter structures. Consequently, potential associations between subcortical alterations and fatigue may have been overlooked.

## Conclusion

Fatigue, a key symptom of PCC, was related to a hypoperfusion of right-hemispheric occipital areas in a prospective cohort of severely affected PCC-patients. This suggests a possible pathophysiological link between fatigue and hypoperfusion of right-hemispheric occipital areas.
